# Functional analysis of the Candida albicans *ECE1* Promoter

**DOI:** 10.1128/spectrum.00253-23

**Published:** 2023-02-14

**Authors:** Enrico Garbe, Nadja Thielemann, Sina Hohner, Animesh Kumar, Slavena Vylkova, Oliver Kurzai, Ronny Martin

**Affiliations:** a Septomics Research Center, Friedrich Schiller University and Leibniz Institute for Natural Product Research and Infection Biology – Hans Knöll Institute, Jena, Germany; b Institute for Hygiene and Microbiology, University of Würzburg, Würzburg, Germany; c Research Group Fungal Septomics, Leibniz Institute for Natural Product Research and Infection Biology – Hans Knöll Institute, Jena, Germany; d National Reference Center for Invasive Fungal Infections, Leibniz Institute for Natural Product Research and Infection Biology – Hans Knöll Institute, Jena, Germany; University of Guelph

**Keywords:** *Candida albicans*, *ECE1*, promoters

## Abstract

The formation of hyphae is a key virulence attribute of Candida albicans as they are required for adhesion to and invasion of host cells, and ultimately deep-tissue dissemination. Hyphae also secrete the peptide toxin candidalysin, which is crucial for destruction of host cell membranes. The peptide is derived from a precursor protein encoded by the gene *ECE1* which is strongly induced during hyphal growth. Previous studies revealed a very complex regulation of this gene involving several transcription factors. However, the promoter of the gene is still not characterized. Here, we present a functional analysis of the intergenic region upstream of the *ECE1* gene. Rapid amplification of cDNA ends (RACE)-PCR was performed to identify the 5′ untranslated region, which has a size of 49 bp regardless of the hyphae-inducing condition. By using green fluorescent protein (GFP) reporter constructs we further defined a minimal promoter length of 1,500 bp which was verified by RT-qPCR. Finally, we identified the TATA element required for the expression of the gene. It is located 106 to 109 bp upstream of the *ECE1* start codon. Our results illustrate that despite a very short 5′ UTR, a relatively long promoter is required to secure *ECE1* transcription, indicating a complex regulatory machinery tightly controlling the expression of the gene.

**IMPORTANCE** In recent years it was shown that secretion of the toxic peptide candidalysin from hyphae of the major human fungal pathogen Candida albicans contributes heavily to its virulence. The peptide is derived from a precursor protein which is encoded by the *ECE1* gene whose transcription is known to be closely associated with formation of hyphae. Here, we used a GFP reporter system to determine the length of the *ECE1* promoter and were able to show that it has a minimal size of 1,500 bp. Surprisingly, the gene has a very short 5′ UTR of only 49 bp. In accordance with this, the TATA element required for transcription is located 106 to 109 bp upstream of the start codon. This indicates that *ECE1* expression is controlled by a very long promoter allowing a complex network of transcription factors to contribute to the gene’s regulation.

## INTRODUCTION

Candidalysin is a cytolytic peptide toxin secreted from hyphae of the opportunistic human fungal pathogen Candida albicans (1). Initially, it was observed that the peptide causes necrotic damage of infected human epithelial cells (1–3), a process which is required for consequent translocation through intestinal barriers (4). Based on these initial findings it was later discovered that candidalysin influences a variety of immunoregulatory mechanisms, including the activation of the NLRP3 inflammasome (5, 6), neutrophil recruitment (7, 8), and release of the proinflammatory cytokines IL-17, IL-36, IL-1-α, IL-1-β, and IL-8 (9, 10). In infected oral epithelial cells, it also triggers the phosphorylation of the epidermal growth factor receptor (EGFR) and activation of the Eph2-EGFR signaling pathway (11, 12). In addition, candidalysin can induce alarmin and antimicrobial peptide release in epithelial cells (13).

Aside from these distinct effects in the interaction between fungal and immune cells, candidalysin participates in more complex relations between the host and C. albicans. Recent works revealed its contributions to the development of allergic airway disease (14) and alcoholic hepatitis (15). The colonization of the gut by C. albicans is controlled by the adaptive immune system (16), yet this interplay also includes a modulation of Th17 cell response by the fungus also present in other host niches (17). Particularly, candidalysin seems to contribute to the establishment of C. albicans commensalism in the gut (18). Further, candidalysin can also be neutralized by serum albumin (19). This peptide is derived from a precursor protein encoded by the C. albicans *ECE1* gene, which is heavily processed in a Kex2/Kex1-dependent manner, resulting in several peptides of which only candidalysin seems to act as a toxic peptide (1, 20–22). Orthologs of this gene are only found in C. dubliniensis and C. tropicalis which are the closest relatives of C. albicans, but no other members of the CTG clade (20).

*ECE1* mRNA is the most abundant one in C. albicans hyphae but is hardly found in yeast cells (23). In the past, several transcription factors were identified which contributed to the complex regulation of the gene in response to different environmental stimuli. Most of these regulators were also shown to be involved in the control of fungal morphology, underlining the tight link between yeast-to-hypha transition and *ECE1* regulation (23–30). The gene also belongs to the small group of core filamentation response (CFR) genes which are always induced during hyphal growth, independent from the hyphae-inducing stimulus (23). Seven of these CFR genes are characterized by large 5′ intergenic regions (IR) of 2,000 bp and more (23). These extended intergenic regions might be suggestive of complex regulatory mechanisms for those genes, which are often linked to fungal morphology and virulence (31, 32). So far, for all but one gene (*ALS3*) the exact length of the promoters is unknown (33). Long upstream intergenic regions can contribute to fine-tuning of the transcriptional and translational control in C. albicans, which was previously shown for *UME6*. Here, the large 5′ untranslated region (5′ UTR) can inhibit translation of the mRNA (34). It is likely that these long intergenic regions upstream of the CFR genes are likewise linked to their transcriptional control. As seen in the current assembly 22 of the C. albicans genome, not only CFR genes are characterized by such long upstream regions but also morphology-associated transcriptional regulator genes like *AHR1*, *BRG1*, *EFG1*, and *NRG1* (35).

Here, we present a functional analysis of the 5′-intergenic region of the C. albicans *ECE1* gene. Via green fluorescent protein (GFP) reporter constructs we defined a minimal promoter required for full-level expression of *ECE1*. Further we utilized 5′ rapid amplification of cDNA ends (RACE)-PCR to identify the length of the 5′ UTR and located the central TATA regulatory element.

## RESULTS

### Defining the 5′ intergenic region of *ECE1*.

**FIG 1:**
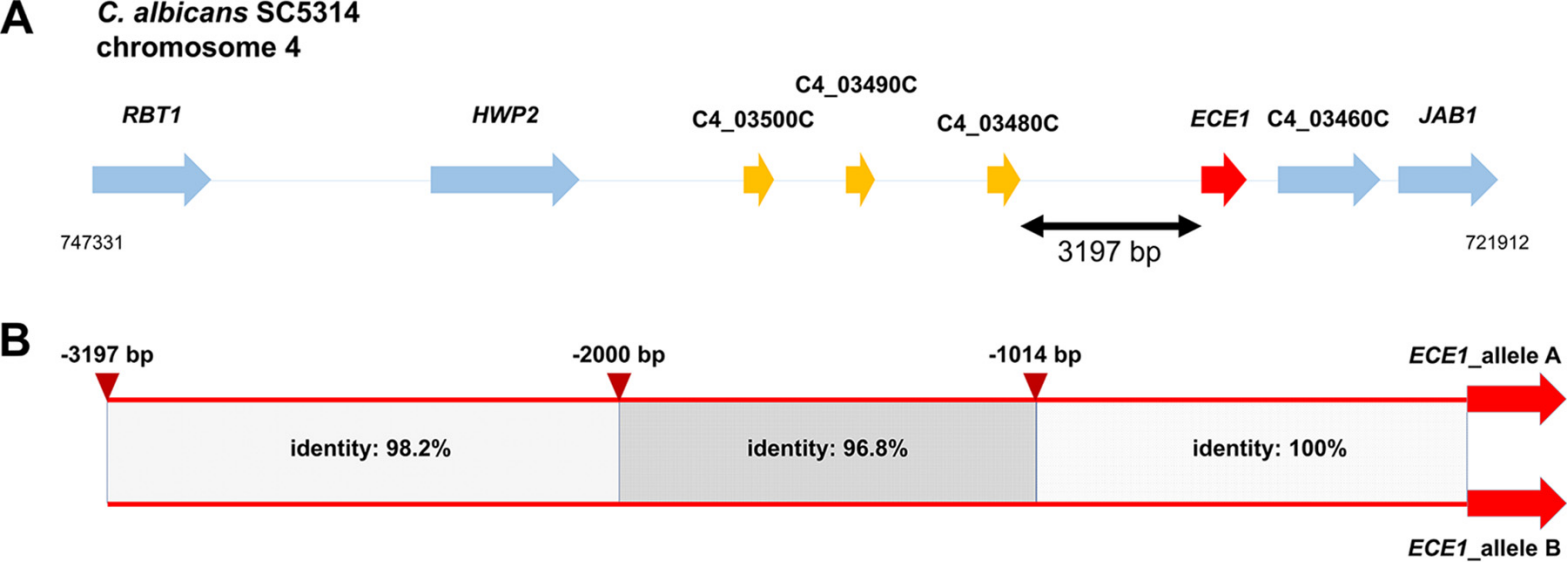
Features of the C. albicans *ECE1* gene locus. (A) Location of *ECE1* and its neighboring genes on chromosome 4 of C. albicans SC5314 according to the Candida Genome Database. (B) Comparison of the 5′ intergenic region (IR) of *ECE1* alleles A and B. Identities between both sequences are shown.

*ECE1* is located on chromosome 4. The current assembly of the of the C. albicans genome shows three small open reading frames (ORFs) upstream of *ECE1*. All of them have a size of approximately 500 bp and are annotated as C4_03480C (588 bp), C4_03490C (525 bp), and C4_03500C (552 bp). A gene with verified function, *HWP2*, is located upstream of them (Fig. 1 A). There is currently no predicted or experimentally verified function for any of the three small. As a basic transcription was described for all of them, they might have a yet unknown biological relevance (31). According to these annotations, we defined the 5′ intergenic region of *ECE1* as the distance between the start codon of the gene and the stop codon of C4_03480C/orf.19.3375, resulting in a size of 3,197 bp (Fig. 1A). This putative promoter region contains allele-specific sequence differences. The first 1,000 bp upstream of the *ECE1* start codon are identical, while most of the differences can be detected between 1,000 and 2,000 bp upstream of ATG (Fig. 1B). Here, the identity between the two alleles is only 96.8%. The region between 2,000 and 3,197 bp upstream of the start codon shares 98.2% identity (Fig. 1B).

### The size of the 5′ untranslated region is independent from the hyphal growth stimulus.

**FIG 2:**
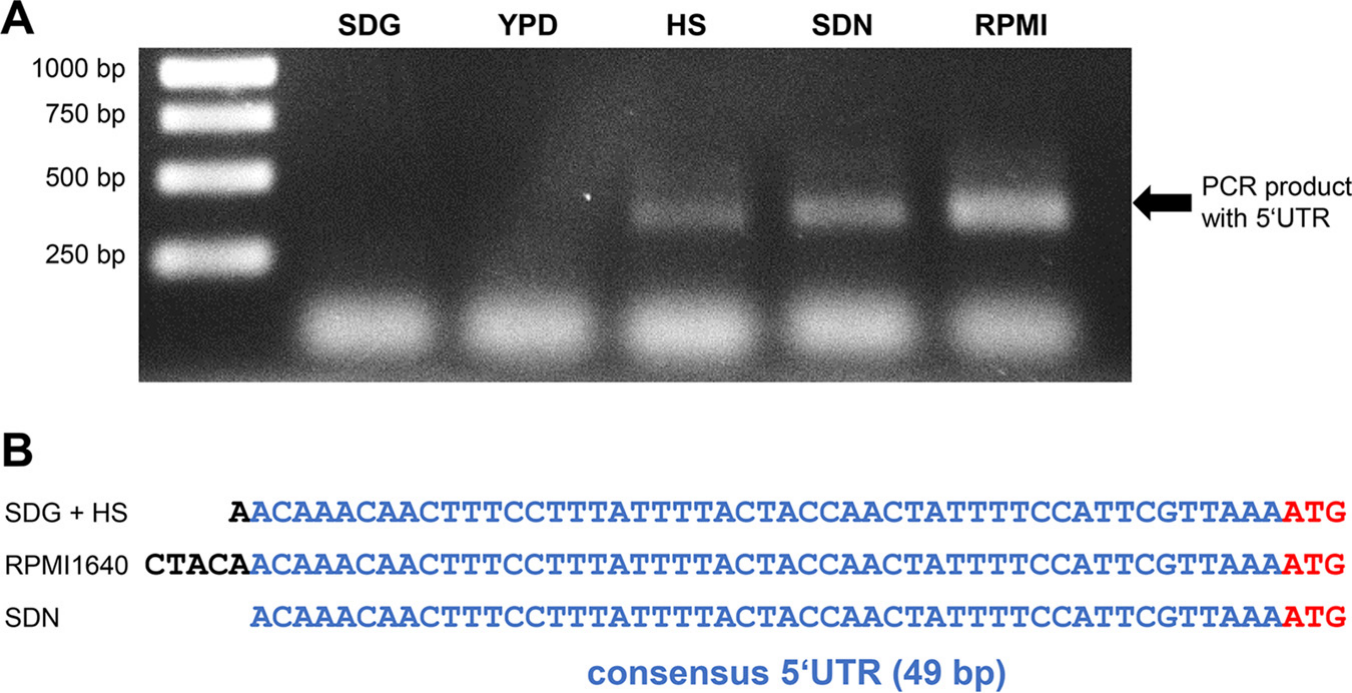
Determination of the 5′ untranslated region (UTR) of the *ECE1* mRNA. 5′ RACE-PCR was performed with RNA from yeast-promoting media (YPD and SDG) and hyphae-inducing media (SDG + human serum [HS], SDN and RPMI). (A) PCR fragments after separation on an 1% agarose gel. These fragments contain the 5′ UTR. (B) PCR fragments from hyphae-inducing conditions were sequenced and aligned. The consensus 5′ UTR sequence is shown in blue; the start codon of *ECE1* is marked in red.

RNA-seq data from previous studies and this work revealed a short size of approximately 50 bp for the *ECE1* 5′ UTR (35, 36). We used 5′ RACE-PCR to verify these observations and analyze the length of the 5′ UTR under three different hyphae-inducing conditions: (i) transfer from SDG minimal medium to SDG with 10% human serum; (ii) transfer from SDG medium to SD medium with *N*-acetylglucosamine as carbon source instead of glucose; and (iii) transfer from yeast extract-peptone-dextrose (YPD) to RPMI 1640 medium. The 5′ RACE-PCR was performed after 60 min of incubation. A PCR product corresponding to the 5′ UTR was neither observed in YPD nor synthetically defined glucose medium (SDG) medium, which was expected as C. albicans exclusively grows in yeast form in both media (Fig. 2). A 5′ UTR could be detected in all three hyphae-inducing media with a consistent size. The consensus 5′ UTR was 49 bp (Fig. 2). This verified the length of the *ECE1* 5′ UTR as short and independent from the environmental stimulus that triggers filamentation.

### Identification of the promoter length with a GFP reporter system.

**FIG 3:**
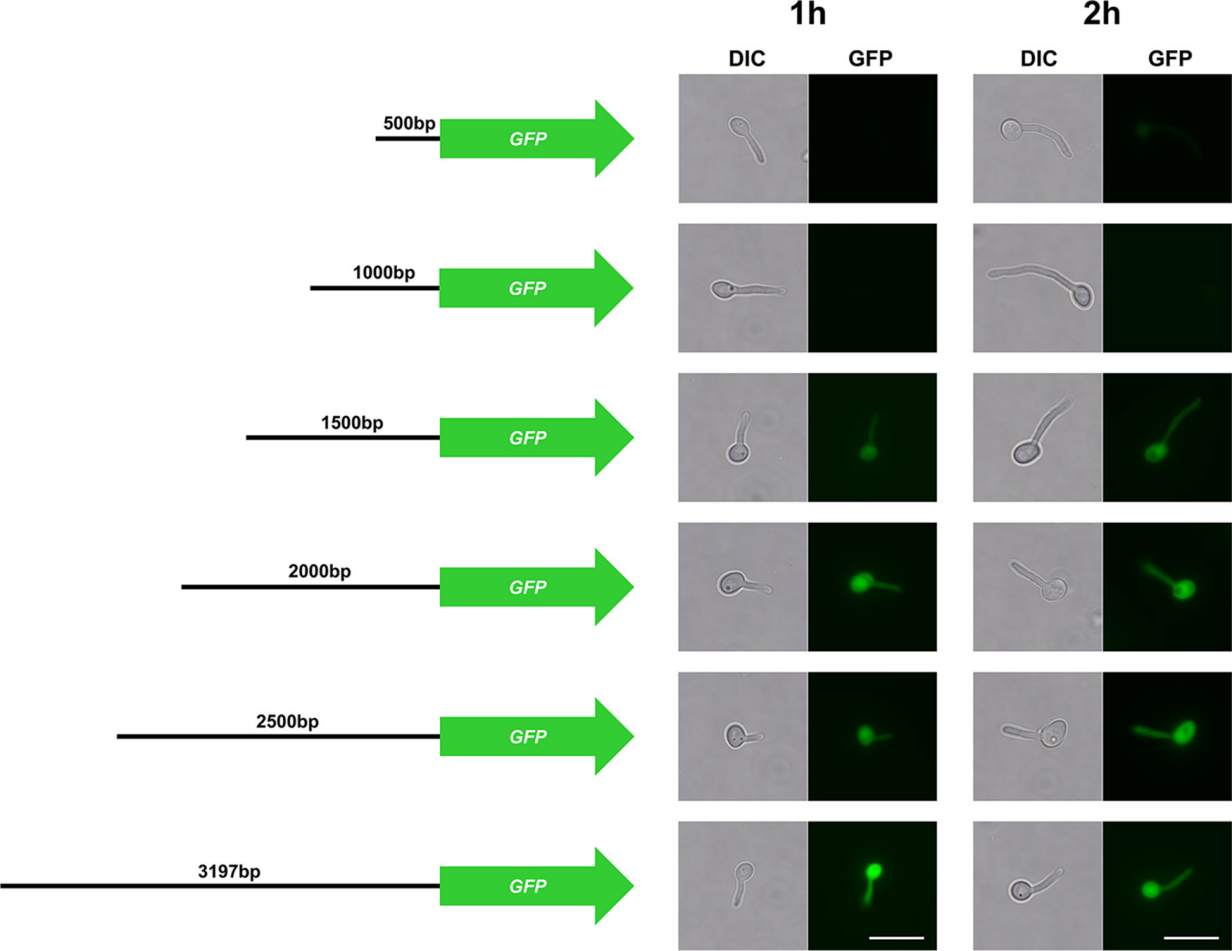
Characterization of the *ECE1* promoter with a GFP reporter system. Truncated versions of the 5′ intergenic region of *ECE1* were fused to *GFP* as indicated. The resulting GFP reporter strains were grown in SDG medium with 10% human serum for 1 and 2 h prior to microscopy. Pictures were taken from the DIC and GFP channel. Scale bar: 10 μm.

Next, we wanted to identify the actual length of the *ECE1* promoter in the 5′ intergenic region and the minimal sequence length required for full-level expression. For this purpose, we established a GFP reporter system. At first, truncated versions of the 5′ intergenic region upstream of the *ECE1* starting codon with the sizes 500 bp, 1,000 bp, 1,500 bp, 2,000 bp, 2,500 bp, and 3,000 bp were fused to *GFP*. Sequences of the truncated *ECE*1 5′ IR versions were identical to the allele A of the *ECE1* gene locus. The resulting constructs were integrated into the transcriptionally neutral *NEUT5L* locus of wild-type strain SC5314. The mutants were then monitored for GFP signals upon hyphae-induction. No GFP signal was observed for strains carrying the pECE1_500_-GFP and the pECE1_1000_-GFP constructs after 1 or 2 h of hyphal growth (Fig. 3). For constructs with 1,500 bp 5′ IR or longer, we noticed GFP fluorescence after 1 and 2 h of hyphae induction (Fig. 3). Therefore, we concluded that GFP expression required at least 1,500 bp of the 5′ intergenic region, while only the first 1,000 bp are insufficient to elicit expression.

**FIG 4:**
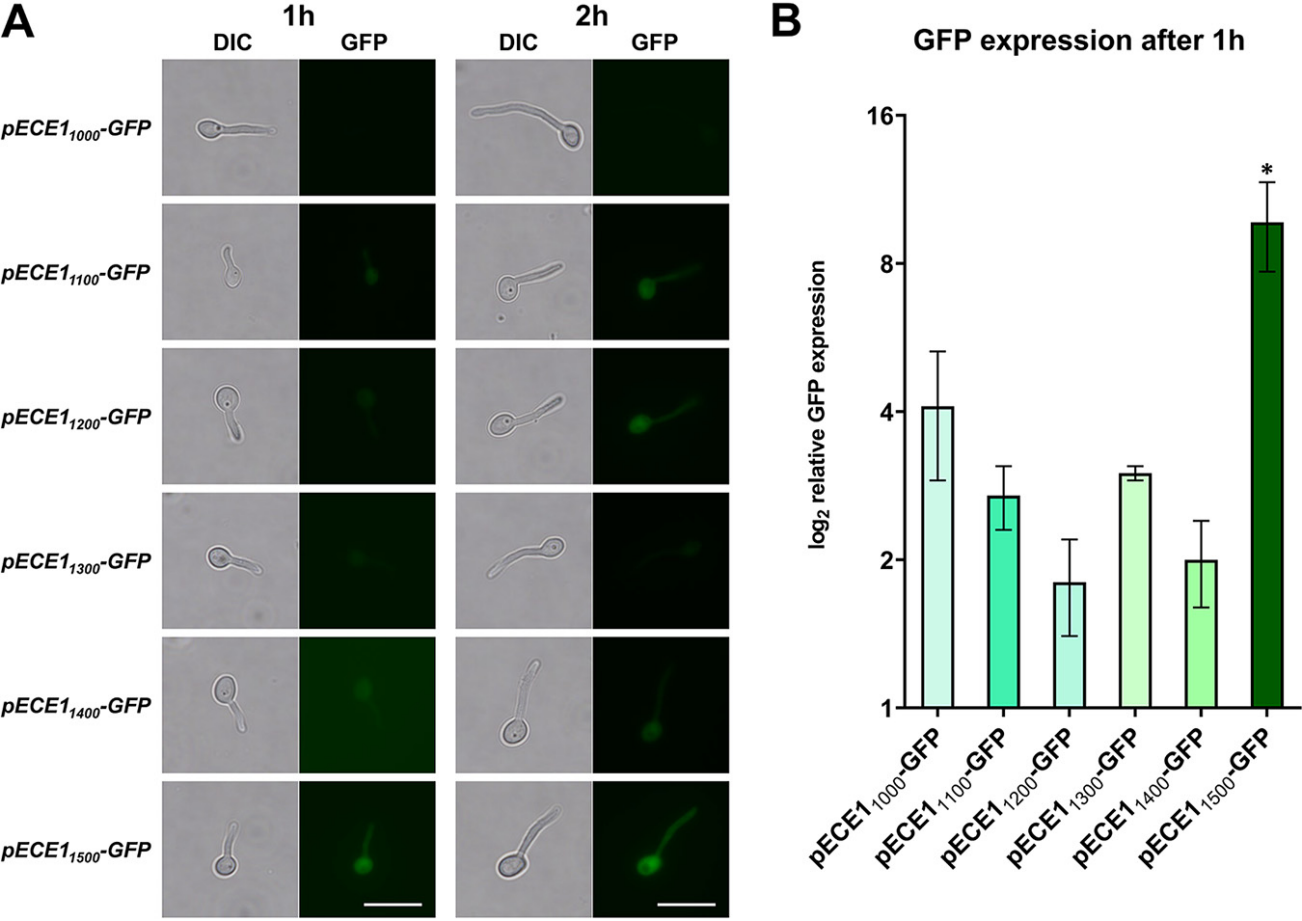
A minimal promoter length of 1,500 bp is required for *ECE1* transcription. The 5′ IR of *ECE1* of the indicated length between 1,100 and 1,400 bp was fused to GFP. (A) Cells of the indicated strains were grown in SDG medium with 10% human serum for 1 and 2 h prior to microscopy. Pictures were taken from the DIC and the GFP channel. Scale bar: 10 μm. (B) GFP expression measured by RT-qPCR from total RNA of cells carrying the indicated constructs. Strains were incubated in SDG medium with 10% human serum for 1 h. GFP expression is shown relative to GFP expression under the *ADH1* promoter in a strain cultured in the same condition. Asterisks mark significant differences in gene expression (*P* ≤ 0.05 in a two-tailed, unpaired Student’s *t* test).

According to these observations, the region between 1,000 and 1,500 bp upstream of the *ECE1* start codon appears crucial for full expression of the gene. To further specify this, we next designed *ECE1* promoter-GFP constructs of 1,100-, 1,200-, 1,300-, and 1,400-bp length (Fig. 4A). Surprisingly, no GFP fluorescence was detected in strains which carried these constructs. Only after 2 h, faint GFP signals were visible in some but not all cells (Fig. 4A). These observations were verified by RT-qPCR results, where *GFP* under the control of less than 1,500 bp of the 5′ intergenic region had decreased expression compared to the pECE1_1500_ construct (Fig. 4B). Taken together, we defined 1,500 bp as the minimal length of the *ECE1* promoter.

### Identification of the TATA box required for the activation of *ECE1* expression.

**FIG 5:**
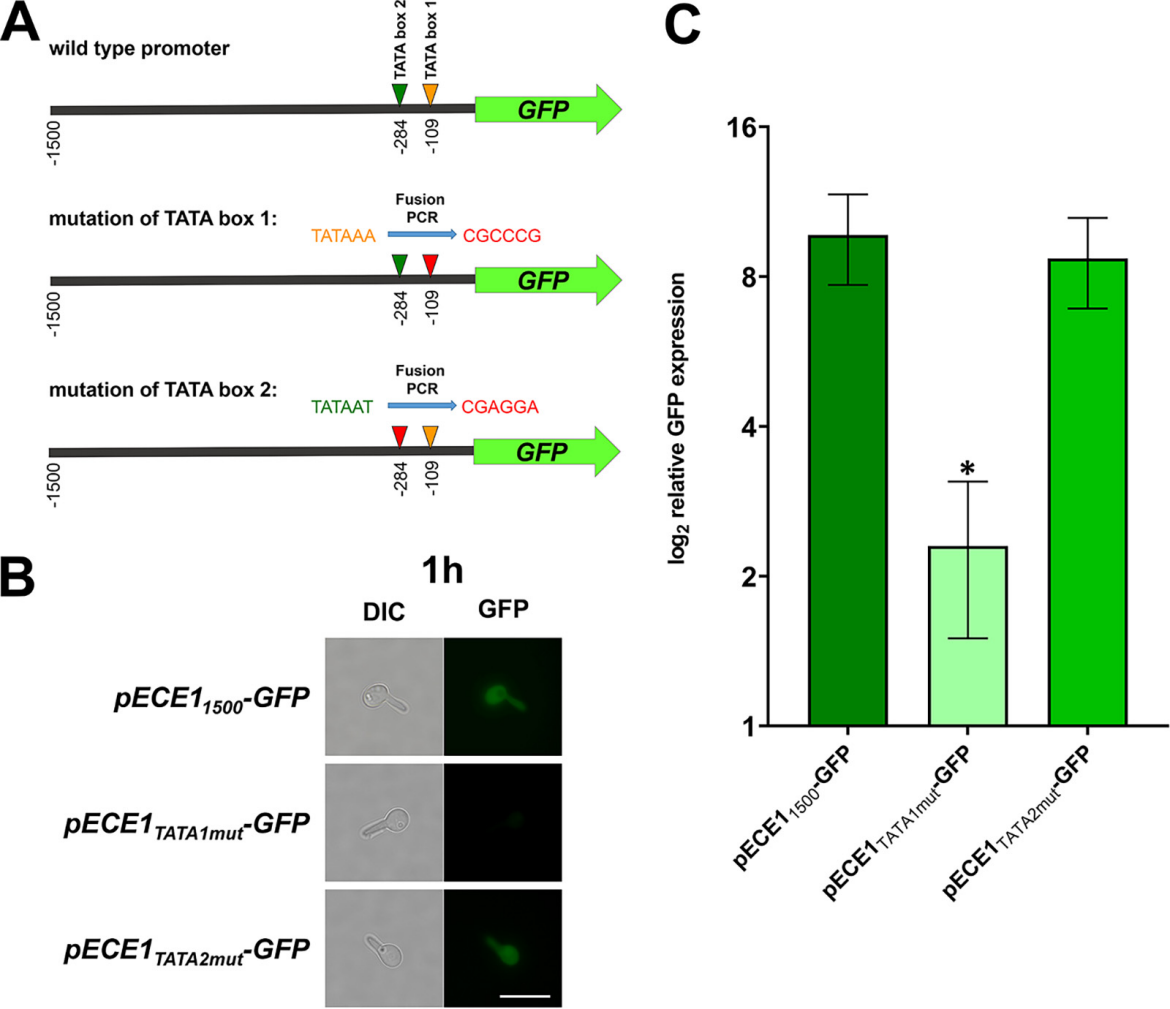
Identification of the TATA element required for *ECE1* transcription. (A) The promoter of *ECE1* contains two TATA elements which might be required for transcription of the gene. They are marked by triangles. Both TATA elements were replaced by non-TATA sequences by fusion PCR. The resulting promoter constructs were then cloned into the GFP reporter system and integrated into C. albicans SC5314. (B) The resulting mutants were grown for 1 h in SDG with 10% human serum at 37°C prior to fluorescence microscopy. Pictures from the GFP and the DIC channels as well as an overlay of both channels are shown. Scale bar: 10 μm. (C) Total RNA was isolated at the same time points and used for RT-qPCR. GFP expression was normalized against GFP under the control of the *ADH1* promoter. The log_2_ values of the relative gene expression are displayed. Asterisks mark significant differences in gene expression (*P* ≤ 0.05 in a two-tailed, unpaired Student's *t* test).

The previous experiments in this study showed that the minimal length of the *ECE1* promoter is 1,500 bp and the size of the consensus 5′ UTR is 49 bp. Within this range, we found two TATA elements which are the most likely candidates for *ECE1* activation. The first element was found 109 bp and the second element 284 bp upstream of the starting codon (Fig. 5A). To study the influence of both TATA elements on *ECE1* transcription we used PCR-based site directed mutagenesis within the pECE1-GFP construct, resulting in a loss of the TATA elements in the respective promoters (Fig. 5A).

After initiation of hyphal growth, the GFP reporter strain with the nonmutated minimal *ECE1* promoter showed a bright fluorescence signal (Fig. 5B). The mutation of the first TATA element resulted in a complete loss of GFP fluorescence (Fig. 5B), while the mutation of the second TATA element resulted in less intense GFP signal compared to the wild-type promoter (Fig. 5B). Via RT-qPCR, we confirmed the significantly lower GFP expression due to mutation of the first TATA element (Fig. 5C). However, GFP expression was not significantly different from the wild-type promoter when the second TATA element was mutated (Fig. 5C).

## DISCUSSION

Unusual long upstream intergenic regions are commonly found in C. albicans genes involved in processes such as adhesion, hyphal morphogenesis, biofilm formation, or white-opaque switching. They often correlate with 5′ UTRs of more than 500 bp (31, 32). These 5′ UTRs are sometimes involved in the regulatory postranscriptional regulatory mechanisms, as shown for the transcription factor gene *UME6* (34). During yeast growth, translational efficiency was found to be suppressed through this secondary structure within the 5′ UTR. Once this region was deleted, protein levels of Ume6 increased, but abundance of the according mRNA remained the same (34). Therefore, one could speculate that long 5′ intergenic regions of the virulence-related and hyphae-associated CFR genes contribute to their complex expression regulation.

Thus, we were interested if a similar mechanism contributes to the regulation of the *ECE1* gene. Its 5′ IR has a size of 3,197 bp which is well above the average size of IR found in the C. albicans genome (23, 31). However, in sharp contrast the 5′ UTR of *ECE1* is notably small. In accordance with previous works, we identified a consensus size of 49 bp in three different filament-inducing media (31, 37). This is well below the mean length of C. albicans 5′ UTRs which is 88 bp (32). Fittingly, we identified an essential TATA element, located 109 bp upstream of the start codon. This distance is within a range that is normal for yeasts (38). Loss of function mutations of this element lead to absent fluorescence in a GFP reporter system, indicating that this TATA box is central for activation of the *ECE1* transcription. Yet, the potential role of the second, more upstream TATA element is not fully clear, as the results of the GFP reporter strains and RT-qPCR validation were ambiguous (Fig. 5). The role of the TATA elements might be different under other hyphae-inducing or environmental conditions. However, as the 5′ UTR size is almost identical for hyphae induction by serum, neutral pH, and *N*-acetylglucosamine, we propose that the -109 TATA element is the essential one for *ECE1* transcription.

Based on our *GFP* reporter experiments with truncated versions of the 5′ intergenic region of *ECE1*, we verified that the minimal size for full-level expression is 1,500 bp and likely represents the core promoter. With this size, the minimal *ECE1* promoter is considerably longer than the average C. albicans promoter, which has a mean length of 623 bp (39).

Surprisingly, we observed no fluorescence for promoter constructs with less than 1,500 bp, indicating that a critical region must be located between 1,400 and 1,500 bp upstream of the start codon. Such critical regions were also identified for *ALS3* and *HWP1*, two other CFR genes (33, 40, 41). In both cases, the regions required for gene activation under hyphal growth conditions are located more than 1,000 bp upstream of the start codon, similar to *ECE1* (33, 41). It was already speculated for *ALS3* that the more distant activation regions might contribute to an enhanced expression of the gene while an activation region closer to the TATA box is required for basic transcription (33).

An obvious explanation of the nature of the critical element located between 1,400 and 1,500 bp of the 5′ IR of *ECE1* could be transcription factor binding sites, required for gene induction upon hyphae-induction. Putative binding sites of transcriptional regulators were previously mapped to the 5′ IR of *ECE1* (30). According to this mapping, the minimal promoter of *ECE1* contains putative binding sites for the transcription factors Ahr1, Bcr1, Brg1, Efg1, Fkh2, Mcm1, Ndt80, Nrg1, and Ume6 (30). However, physical evidence for binding was so far only provided for Ahr1 (30). Interestingly, none of these regulators putatively bind to the region between 1,400 and 1,500 bp upstream of the start codon. Thus, potential binding events and the identity of the regulatory proteins remain elusive.

We previously showed that Tup1 is essential for efficient repression and activation of *ECE1* (30). So far, localization of the Tup1 binding to the *ECE1* promoter is unknown. It is also unclear if there is a direct binding of this factor or one mediated by cofactors like Nrg1. The restriction of the minimal promoter might ease future experiments as they can focus on this region to analyze the physical binding of transcriptional regulators.

According to the current assembly of the C. albicans genome, most of the other CFR genes possess 5′ IRs of more than 2,000 bp (35). Interestingly, the genome analysis by Bruno et al. (31) indicated that the size of the 5′ UTRs of *ALS3*, *HWP1*, and *IHD1* was also only around 50 bp, similar to the 5′ UTR of *ECE1*. It might be that these virulence-associated genes spare the possibility of 5′ UTR-mediated regulation. Considering their central functions, high and quick expression in response to environmental stimuli might be ensured in a manner of an all or nothing expression. Future experiments will elucidate if *ECE1* and CFR genes are indeed primarily transcriptionally regulated and if posttranslational mechanisms contribute to their abundance.

## MATERIALS AND METHODS

### C. albicans strains, growth conditions, and media.

In this study, we used the wild-type strain SC5314 (36) or derivates of it. All strains are listed in Table 1. They were routinely grown in YPD (20 g/L glucose, 10 g/L yeast extract, 20 g/L casein peptone) or SDG (20 g/L glucose, 6.7 g/L yeast nitrogen base without amino acids) medium at 37°C. For solid medium, 20 g/L agar were added. Hyphal growth was induced by the addition of 10% human serum (Sigma-Aldrich) to SDG medium.

**TABLE 1:** C. albicans strains used in this study

Strain	Genotype	Source
SC5314	Wild type	(37)
pADH1-GFP	SC5314, *ADH1/adh1::GFP-SAT1*	(42)
pECE1-GFP	SC5314, *ECE1/ece1::GFP-SAT1*	(1)
pECE1_500_-GFP	SC5314, *NEUT5L/neut5l::pECE1_500_-GFP-SAT1*	This work
pECE1_1000_-GFP	SC5314, *NEUT5L/neut5l::pECE1_1000_-GFP-SAT1*	This work
pECE1_1500_-GFP	SC5314, *NEUT5L/neut5l::pECE1_1500_-GFP-SAT1*	This work
pECE1_2000_-GFP	SC5314, *NEUT5L/neut5l::pECE1_2000_-GFP-SAT1*	This work
pECE1_2500_-GFP	SC5314, *NEUT5L/neut5l::pECE1_2500_-GFP-SAT1*	This work
pECE1_3000_-GFP	SC5314, *NEUT5L/neut5l::pECE1_3000_-GFP-SAT1*	This work
pECE1_1100_-GFP	SC5314, *NEUT5L/neut5l::pECE1_1100_-GFP-SAT1*	This work
pECE1_1200_-GFP	SC5314, *NEUT5L/neut5l::pECE1_1200_-GFP-SAT1*	This work
pECE1_1300_-GFP	SC5314, *NEUT5L/neut5l::pECE1_1300_-GFP-SAT1*	This work
pECE1_1400_-GFP	SC5314, *NEUT5L/neut5l::pECE1_1400_-GFP-SAT1*	This work
pECE1_T1mut_-GFP	SC5314, *NEUT5L/neut5l::pECE1_T1mut_-GFP-SAT1*	This work
pECE1_T2mut_-GFP	SC5314, *NEUT5L/neut5l::pECE1_T2mut_-GFP-SAT1*	This work

### Construction of plasmids for the p*ECE1*-*GFP* reporter system.

Bluescript pSK (Agilent Technologies; Table 2) was used as a template for all constructed plasmids. At first, the C. albicans *ACT1* terminator was amplified from genomic C. albicans SC5314 DNA with the oligonucleotide primers 5′CaACT1term-EcoRI and 3′CaACT1term-SpeI (Table 3). The terminator was then cloned into pSK via EcoRI/SpeI. Second, C. albicans optimized *GFP* was amplified from pSK-CaGFP (42) with primers 5′GFP-XhoI and 3′GFP-EcoRV and then cloned into pSK with the *CaACT1* terminator. In a third step, the *CaSAT1* gene, including the *CaACT1* promoter, *CaSAT1* ORF and *CaURA3* terminator, was excised from pSK-CaGFP with NotI and then cloned into NotI-restricted pSK with *CaACT1* terminator and GFP. In a next step, the 5′ homology region to the *NEUT5L* locus was amplified with primers 5′NEUfwd-ApaI and 5′NEUrev-ANP-XhoI. The latter oligonucleotide contained additional restriction sites for *Asc*I and *Nar*I. The resulting PCR fragment was cloned via ApaI and XhoI into ApaI/XhoI-restricted pSK with GFP, *CaACT1* terminator and *SAT1*. The 3′ homology region of *NEUT5L* was then amplified with primers 3′NEUfwd-SacII and 3’′NEUT5L-SacI-PacI and then cloned via *Sac*II/SacI into *Sac*II/SacI-restricted pSK with the other fragments. Finally, the first 500 bp upstream of the *ECE1* start codon were amplified with oligonucleotide primers 5′ECE1prom500-AscI and 3′ECE1prom-XhoI. The amplified 500-bp fragment was then cloned into pSK-GFP-SAT1 with NEUT5L homology regions via *Asc*I/XhoI. The resulting plasmid was then called pSK-pECE1_500_-GFP-SAT1 (Table 2). All other plasmids with the truncated versions of the C. albicans *ECE1* promoter were derived from this plasmid by replacing the 500-bp fragment against PCR products of 3′ECE1prom-XhoI and 5′ECE1promX-AscI primers. X stands for the respective sizes of the promoter fragments. Cloning was performed by using the *Asc*I and XhoI restriction sites. All constructed plasmids are listed in Table 2.

**TABLE 2:** Plasmids used in this study

Plasmid	Features	Source
pSK bluescript	Bluescript plasmid with Amp^R^	Agilent
pSK-pECE1_500_-GFP-SAT1	pSK, pECE1_500_ fused to GFP, SAT1 as selection marker, and homology regions for integration into CaNEUT5L locus	This work
pSK-pECE1_1000_-GFP-SAT1	pSK, pECE1_1000_ fused to GFP, SAT1 as selection marker, and homology regions for integration into CaNEUT5L locus	This work
pSK-pECE1_1500_-GFP-SAT1	pSK, pECE1_1500_ fused to GFP, SAT1 as selection marker, and homology regions for integration into CaNEUT5L locus	This work
pSK-pECE1_2000_-GFP-SAT1	pSK, pECE1_2000_ fused to GFP, SAT1 as selection marker, and homology regions for integration into CaNEUT5L locus	This work
pSK-pECE1_2500_-GFP-SAT1	pSK, pECE1_2500_ fused to GFP, SAT1 as selection marker, and homology regions for integration into CaNEUT5L locus	This work
pSK-pECE1_3000_-GFP-SAT1	pSK, pECE1_3000_ fused to GFP, SAT1 as selection marker, and homology regions for integration into CaNEUT5L locus	This work
pSK-pECE1_T1Mut_-GFP-SAT1	pSK, pECE1_1500_ with mutated TATA box 1 (−109), fused to GFP, SAT1 as selection marker, and homology regions for integration into CaNEUT5L locus	This work
pSK-pECE1_T2Mut_-GFP-SAT1	pSK, pECE1_1500_ with mutated TATA box 2 (−284), fused to GFP, SAT1 as selection marker, and homology regions for integration into CaNEUT5L locus	This work

**TABLE 3:** Oligonucleotide primers used in this study

Name	Sequence in 5′ to 3′ direction^*a*^
5′GFP-XhoI	AGCTctcgagATGAGTAAGGGAGAAGAACTTTTCACTGGA
3'GFP-EcoRV	AGCTgatatcTTATTTGTATAGTTCATCCATGCCATGTGT
5′CaACT1term-EcoRI	AGCTgaattcGAGTGAAATTCTGGAAATCTGGAAATCT
3'CaACT1term-SpeI	AGCTactagtTAGATTATGGTCGACATTTTATGATGGAAT
5′NEUfwd-ApaI	ACTGgggcccGTAATTGTAGTAAGAATGACAAGTATCAG
5'NEUrev-ANP-XhoI	ACTGctcgagttaattaaggcgccggcgcgccGGAAGGACGATGAAGGAGAGAAAG
3′NEUfwd-SacII	ACTGccgcggTAAACAAGTGGTATTCAAGCACAATTC
3'NEUT5L-SacIPacI	GCTGgagctcttaattaaTAACCCACTGAATTCTACATCGAAC
3'ECE1prom-XhoI	TCATctcgagTTTAACGAATGGAAAATAGTTGGTAGTAAAATAAAGG
5′ECE1prom500-AscI	TTCCggcgcgccGTCATTTGTAGGATTTTCAGCAGAACA
5′ECE1prom1000-AscI	TTCCggcgcgccAGCGAAACATTTTTTTTTTTCAACGGCTC
5′ECE1prom1500-AscI	TTCCggcgcgccACCCTAGTAATTATATGAAACATGCCCA
5′ECE1prom2000-AscI	TTCCggcgcgccAACATTAACGACGCAAAATACAAACTTGGT
5′ECE1prom2500-AscI	TTCCggcgcgccGTAAGCATTTTGGAGTAATACCATAGTTG
5′ECE1prom3000-AscI	TTCCggcgcgccGCAAATAGAATTGTTCTATTGCTTAGCTTTAG
R1-CaACT1	TCAGACCAGCTGATTTAGGTTTG
R2-CaACT1	GTGAACAATGGATGGACCAG
R1-CaECE1	ATCGAAAATGCCAAGAGAG
R2-CaECE1	AGCATTTTCAATACCGACAG
R1-CaGFP	CTGAAGTCAAGTTTGAAGGTGATAC
R2-CaGFP	GCAGATTGTGTGGACAAGTAATG
GFP veri rev	TGATCTGGGTATCTCGCAAAGCAT

a Lowercase: restriction sites.

### Construction of plasmids for TATA element mutations.

For the identification of the TATA element required for transcription of *ECE1*, we have used fusion PCRs to construct pECE1-GFP reporter cassettes carrying loss of function mutations in either TATA box 1 or 2. We have used the pSK-pECE1_1500_-GFP plasmid as a template to amplify a promoter construct which is suitable for *GFP* expression.

For TATA box 1, which is located 109 bp upstream of the *ECE1* start codon, we first amplified a 128-bp PCR product with primers pECE1 TATA1fwd and 3′ECE1prom-XhoI. Second, primers 5′ECE1prom1500-AscI and pECE1-TATA1 rev were used to amplify a 1,433-bp PCR product. Primers pECE1-TATA1fwd and pECE1-TATA1rev provide a 24-bp overlap which would introduce a loss of function mutation from TATAAA to CGCCCG of the TATA box 1. The overlap was used for the following fusion PCR to construct a 1,500-bp promoter construct without TATA box 1. Both primary PCR products were used as a template for fusion PCR with the primers 5′ECE1prom1500-AscI and 3′ECE1prom-XhoI to amplify a 1,536-bp product.

Mutation of TATA box 2 was performed in the same way. It is located 284 bp upstream of the *ECE1* start codon and we first amplified a 302-bp PCR product with primers pECE1-TATA2fwd and 3′ECE1prom-XhoI. A second PCR product of 1258 bp was then amplified with the primers 5′ECE1prom1500-AscI and pECE1-TATA2 rev. Primers pECE1-TATA2fwd and pECE1-TATA2rev provide a 24-bp overlap which would introduce a mutation from TATAAT to CGAGGA at the site of TATA box 1. Both PCR products containing this overlap were then used as a template for fusion PCR with the primers 5′ECE1prom1500-AscI and 3′ECE1prom-XhoI.

The fusion PCR products containing either the TATA box 1 or the TATA box 2 mutations were finally restricted with *Asc*I and XhoI and cloned into an *Asc*I/XhoI digested pECE1_500_-GFP plasmid. The integration of the mutated TATA box was verified by Sanger sequencing (LGC Genomics, Berlin, Germany).

### Construction of C. albicans strains.

The transformation cassettes were excised from the pECE1-GFP plasmids with ApaI/PacI. The fragments were then transformed into C. albicans SC5314 by using the established lithium acetate method (43). Integration of the transformation cassettes into the NEUT5L locus was verified by colony PCR. Oligonucleotide primers used for colony PCRs can be found in Table 3.

### Gene expression analysis.

For the analysis of *GFP* expression, we have used selected C. albicans strains carrying *GFP* under the control of either the *ADH1* promoter or different versions of the *ECE1* promoter. They were first grown in SDG medium overnight at 37°C. From these precultures, 1 × 10^6^ cells/mL were diluted into fresh SDG medium (prewarmed at 37°C) which contained 10% human serum if required. Cells were then grown for 1 or 2 h at 37°C. Afterwards, total RNA was isolated from the harvested cells as previously described (29). Quantitative RT PCR was performed using the Luna universal one-step RT-qPCR kit (New England Biolabs) on a QTower3 (Analytik Jena) with 100 ng/μL RNA. Gene expression of *GFP* under the control of the truncated *ECE1* promoter constructs was normalized against *GFP* under the control of the *ADH1* promoter. Calculation was done using the ΔΔCt method (44). Expression data from biological triplicates were compared with a two-tailed, unpaired Student's *t* test and only differences with a *P* value of ≤0.05 were regarded as statistically significant.

### RACE-PCR.

The size of the 5′ untranslated region of *ECE1* mRNA was determined via 5′ RACE-PCR. For this, we have used the 5′ RACE-PCR kit (Life Technologies, Darmstadt) and gene-specific primers for *ECE1* (Table 3). RACE-PCRs were performed according to manufacturer’s instructions. 1 × 10^6^ cells/mL of C. albicans SC5314 were grown in different yeast- or hyphae-inducing media for 1 h at 37°C. Afterwards total RNA was isolated as described above. A total of 2.5 μg of total RNA isolate were used for the initial cDNA synthesis. PCRs of the nested amplification rounds 1 and 2 were performed using the Q5 High Fidelity 2X master mix (New England Biolabs). Oligonucleotide primer GSP3 contains a XhoI restriction site for the subsequent cloning of the final PCR product into pSK plasmid, allowing Sanger sequencing of the PCR product and identification of the 5′ UTR sequence.

### Fluorescence microscopy.

Fluorescence microscopy was performed with a Nikon Eclipse Ni microscope (Nikon, Germany). Differential interference contrast (DIC) illumination time was 200 ms and for the GFP channel it was 2,000 ms. The same illumination times were used for all samples to guarantee comparable GFP signals.
